# Post-Translational Modifications in Oocyte Maturation and Embryo Development

**DOI:** 10.3389/fcell.2021.645318

**Published:** 2021-06-02

**Authors:** Yu Wu, Mo Li, Mo Yang

**Affiliations:** ^1^Department of Obstetrics and Gynecology, Peking University Third Hospital, Beijing, China; ^2^National Clinical Research Center for Obstetrics and Gynecology, Peking University Third Hospital, Beijing, China; ^3^Medical Center for Human Reproduction, Beijing Chaoyang Hospital, Capital Medical University, Beijing, China

**Keywords:** oocyte maturation, embryo development, PTM, ubiquitination, infertility

## Abstract

Mammalian oocyte maturation and embryo development are unique biological processes regulated by various modifications. Since *de novo* mRNA transcription is absent during oocyte meiosis, protein-level regulation, especially post-translational modification (PTM), is crucial. It is known that PTM plays key roles in diverse cellular events such as DNA damage response, chromosome condensation, and cytoskeletal organization during oocyte maturation and embryo development. However, most previous reviews on PTM in oocytes and embryos have only focused on studies of *Xenopus laevis* or *Caenorhabditis elegans* eggs. In this review, we will discuss the latest discoveries regarding PTM in mammalian oocytes maturation and embryo development, focusing on phosphorylation, ubiquitination, SUMOylation and Poly(ADP-ribosyl)ation (PARylation). Phosphorylation functions in chromosome condensation and spindle alignment by regulating histone H3, mitogen-activated protein kinases, and some other pathways during mammalian oocyte maturation. Ubiquitination is a three-step enzymatic cascade that facilitates the degradation of proteins, and numerous E3 ubiquitin ligases are involved in modifying substrates and thus regulating oocyte maturation, oocyte-sperm binding, and early embryo development. Through the reversible addition and removal of SUMO (small ubiquitin-related modifier) on lysine residues, SUMOylation affects the cell cycle and DNA damage response in oocytes. As an emerging PTM, PARlation has been shown to not only participate in DNA damage repair, but also mediate asymmetric division of oocyte meiosis. Each of these PTMs and external environments is versatile and contributes to distinct phases during oocyte maturation and embryo development.

## Introduction

Oocyte maturation is a unique type of cell division that is different from mitosis. It is defined as the reinitiation and completion of the first meiotic division (Trounson et al., 1998). Oocytes arrest at the germinal vesicle (GV) phase for decades, then when stimulated by a surge of luteinizing hormone (LH), germinal vesicle breakdown (GVBD) occurs with gradual condensation of chromosomes (Motlik and Fulka, 1976; Mattson and Albertini, 1990). When the chromosomes are fully condensed, the oocyte enters the metaphase I (MI) stage. During this stage, chromosomes align on the metaphase plate and spindle microtubules attach to the kinetochores, preparing for chromosome segregation in the following anaphase I. After the exclusion of the first polar body, oocytes arrest at metaphase II (MII) until fertilization (Webster and Schuh, 2017). Any defects in this process may lead to arrest of meiosis and failure of fertilization. Because of the absence of transcriptional activities during oocyte maturation, post-translational modification (PTM) is critical to accomplish oocyte maturation (Chen et al., 2011).

There is also no transcription of the zygote genome at the early stages of embryo development, which begins at fertilization when a haploid oocyte fuses with a sperm. Although the maternal and paternal gametes offer equal genetic materials, early embryo development mainly depends on maternal mRNAs and proteins in the oocyte (Osman et al., 2018). These proteins are mainly activated during fertilization and the maternal-to-zygotic transition (Yartseva and Giraldez, 2015). Zygotic genome activation predominantly occurs at the 2-cell stage in mice, and at the 4-cell stage and 8-cell stage in humans (Xue et al., 2013; Yan et al., 2013; Lee et al., 2014). Because of the inactivation of the zygotic genome during early embryo development, PTM is particularly crucial. Errors of PTM in both oocyte maturation and early embryo development may cause implantation problems, fetal defects, and placental dysfunctions (Samson et al., 2014; Illingworth et al., 2015; Robajac et al., 2016).

Post-translational modifications include phosphorylation, ubiquitination, neddylation, SUMOylation, and PARylation. It is identified that PTMs such as phosphorylation regulate oocyte maturation and embryo development (Nishi and Lin, 2005; Das et al., 2016). But because of sample limitations, most reports about PTMs during oocyte maturation and early embryo development are restricted to zebrafish, *Xenopus laevis* or *Caenorhabditis elegans* eggs. In this review, we will discuss the recent discoveries about PTMs of mammalian oocytes, with a focus on phosphorylation, ubiquitination, SUMOylation, and Poly(ADP-ribosyl)ation (PARylation).

## Phosphorylation Regulates Oocyte Maturation Mainly Through the Mitogen-Activated Protein Kinase Pathway

### Phosphorylation Is a Common PTM in Eukaryotes

Phosphorylation is considered as a common PTM in eukaryotes (Cohen, 2000). Most proteins in mammalian cells are phosphorylated (Olsen et al., 2010); it is estimated that almost 13,000 proteins can be phosphorylated in human (Vlastaridis et al., 2017). Phosphorylation catalyzed by protein kinases is a reversible process that mainly occurs on serine, threonine, and tyrosine residues through the formation of phosphoester bonds. Phosphorylation changes the structures of the substrate proteins, thus allowing proteins to be activated or deactivated, stabilized or destabilized (Cohen, 2002). In addition, phosphorylation can regulate protein localization, interactions with other proteins, and stability by altering the electrostatic environment (Yokoi et al., 1993; Narayanan and Jacobson, 2009). In mammals, phosphorylation is involved in many biological processes such as the cell cycle (Besterman and Schultz, 1990), cell differentiation (Hardwick et al., 2018), and apoptosis (Nakamizo et al., 2008). However, although this PTM is important and ubiquitous, how phosphorylation regulates oocyte maturation and embryo development in mammals is still not well understood. It is speculated that phosphorylation may regulate these processes by regulating proteins involved in chromosome condensation and spindle assembly (Swain et al., 2007; Zhang Y. et al., 2018). Using okadaic acid to inhibit protein phosphatases, chromosome condensation occurred prematurely and pig oocytes entered the GVBD stage early as a result (Sun et al., 2002).

### Histone Phosphorylation Regulates Chromosome Condensation

Chromosome condensation is an essential prerequisite for chromosome segregation during meiosis. Any error of chromosome condensation will lead to an unexpected abnormality of oocyte maturation and oocyte-derived embryonic aneuploidy, resulting in infertility. Histone phosphorylation was first observed as early as the 1960s (Gutierrez and Hnilica, 1967), and it was later found that histones were phosphorylated at phosphor-acceptor sites at the N-terminus (Galasinski et al., 2002). Among histones, phosphorylation of histone H3 is the most well studied in mammals. It is known that chromosome gradually condensed in GVBD stages. Studies have revealed that the activity of histone H3 kinase is low at the GV stage and increases at the GVBD, MI, and MII stages (Bui et al., 2004; Swain et al., 2007). Similarly, phosphorylation of histone H3 at Ser10 is first detected at the GVBD stage and is maintained until the MII stage during mouse oocyte meiosis (Swain et al., 2007). Though there are conflicting reports about whether the phosphorylation of histone H3 is required for chromosome condensation in mitosis (Hendzel et al., 1997; Van Hooser et al., 1998), these researches show that phosphorylated histone H3 is an important regulator of chromosome condensation during oocyte meiosis (Huang et al., 2018). Phosphorylation always occurs at Ser10 and Ser28 of the H3 N-terminal tail and the phosphorylated H3 recruits factors involved in chromosome condensation (Wei et al., 1998, 1999; Nowak and Corces, 2004). Other studies showed that phosphorylated histone H3 was localized close to the chromatin at the GV-stage oocytes and was retained along the entire chromosomes when oocytes entered the MI stage (Wang et al., 2006; Gu et al., 2008). Also, the phosphorylation of histone H3 at Ser28 is triggered at pre-MI during porcine oocyte maturation (Wang et al., 2006; Gu et al., 2008). This further reminds us that phosphorylation of histone H3 plays a key role in chromosome condensation at least. Moreover, rather than Ser28 and Ser10, histone H3 phosphorylation at Thr 3 (H3/Thr3ph) has been shown to be vital for chromosome dynamics during mitosis (Kang et al., 2015), with inhibition of H3/T3ph impairing the segregation of homologous chromosomes (Besterman and Schultz, 1990).

By which pathway does histone H3 regulate the chromosome condensation and segregation? It has been suggested that mitogen-activated protein kinases (MAPKs), also referred to as extracellular-regulated protein kinases (ERKs), mediate histone H3 phosphorylation (Sassone-Corsi et al., 1999). MAPKs are Ser/Thr protein kinases, some of which are involved in mammalian oocyte maturation (Faerge et al., 2001; Su et al., 2002; Prochazka and Nemcova, 2019), and are phosphorylated and activated by MAPK kinases and MAPK kinase kinases after GVBD in mammalian oocytes (Lu et al., 2001; Tan et al., 2001). MAPK3 and MAPK1, also known as ERK1 and ERK2, respectively, are ubiquitously expressed in mammalian tissues as well as in oocytes (Su et al., 2002; Fan et al., 2009). It is known that MAPK3/1 is activated by MEK1/2 (Verlhac et al., 2000). When MAPK1 and MAPK3 are inactivated using a MEK inhibitor, mouse oocytes are blocked from entering the GVBD stage *in vitro* (Su et al., 2002). Granulosa cells have endocrine functions during oocyte maturation. Depletion of *Erk1/2* in ovarian granulosa cells disrupts LH-induced oocyte meiosis, ovulation, and luteinization through its downstream mediator CCAAT/Enhancer-binding protein-β (C/EBPβ) (Fan et al., 2009).

Many studies focus on the regulation of chromosome condensation by phosphorylation. In addition, phosphorylation is speculated to participate in many other structural and functional processes, such as chromosome segregation and spindle arrangement during and after the MII stage. Yet, the mechanisms are not fully understood and need to be further investigated. Phosphorylation is time-dependent and dynamically regulates meiosis (Sun et al., 2002). But the molecular mechanism is still uncertain. Further investigations are expected to bring information on the dynamic changes of proteomics during oocyte maturation and embryo development.

### Environmental Exposure Disrupts Oocyte Maturation Through Phosphorylation

In addition to biological regulation mechanisms, environmental exposure also plays a considerable role in reproductive processes (Patisaul and Adewale, 2009). There are numerous toxins in the environment that influence the oocyte maturation process. One example is bisphenol AF (BPAF), which is widely produced for plastics and epoxy resins and is a ubiquitous toxin in our environment (Villeneuve et al., 2012). Upon the exposure to BPAF, MAPK phosphorylation is disrupted, causing abnormal spindle morphology and disrupted polar body extrusion (Ding et al., 2017). Similarly, treatment with HT-2, zearalenone, or fumonisin B1 causes spindle abnormalities (Zhu et al., 2014, 2016; Hou et al., 2015; Han et al., 2016). These toxins are suggested to mainly affect MAPK phosphorylation. However, the effects of deoxynivalenol to disrupt spindle formation and the interaction between the kinetochore and microtubules are suggested to be mediated by oxidative stress (Lan et al., 2018). These results show that environmental exposure does disrupt oocyte maturation. Though we speculate that it is regulated by phosphorylation, the exact mechanism is still not clear. As environmental problems become more and more intractable all over the world and are known to be related to impaired fertility, developing new targets is increasingly significant for future therapy.

## Ubiquitination Regulates Oocyte Maturation and Embryo Development

### Ubiquitination Is a Three-Step PTM Regulating Protein Degradation

Ubiquitin is a small protein of 8.6 kDa and is universally expressed in eukaryotic cells (Dikic, 2017). Ubiquitination is the process by which ubiquitin is added to target substrates and regulates their degradation. It is known that seven lysine residues (K6, K11, K27, K29, K33, K48, and K63) and an N-terminal methionine (M1) are the sites for ubiquitin to bind with its substrates (Cohen-Kaplan et al., 2016; Akimov et al., 2018). Ubiquitination occurs *via* a three-step cascade sequentially mediated by a ubiquitin-activating enzyme (E1), a ubiquitin-conjugating enzyme (E2), and a ubiquitin ligase (E3) (Behrends and Harper, 2011). These three steps are correspondingly named activation, conjugation, and ligation. Activation, the first step of ubiquitination, is dependent on ATP. E1 binds to ATP and ubiquitin, and activates acyl-adenylation of the ubiquitin C-terminus. During conjugation, which is catalyzed by E2 ubiquitin-conjugating enzymes, the ubiquitin is transferred from the E1 to E2. E2 enzymes contain a highly conserved domain known as the ubiquitin-conjugating catalytic (UBC) domain. Ligation is the final step and is catalyzed by E3 ubiquitin ligases. An isopeptide bond is formed between the glycine residue of the ubiquitin C-terminus and the target protein. There are two major superfamilies of E3s: the RING-domain E3s and homologous to E6-AP C terminus (HECT) E3s. As ubiquitination of proteins occurs universally, thousands of substrates of ubiquitination have been identified to date. In a recent study, Akimov et al. (2018) identified over 63,000 unique ubiquitination sites on 9,200 proteins in human cell lines by mass spectrometry. It is relevant to the regulation of the cell cycle, fertilization, oocyte maturation, and embryo development (Xuan et al., 2018; Zhang Y. et al., 2018).

**FIGURE 1:**
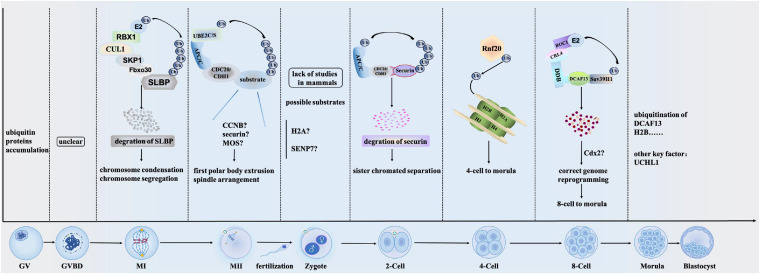
Overview of the main ubiquitination mechanisms during oocyte maturation and preimplantation embryo development. Ubiquitination regulates protein stability during almost all stages of oocyte maturation and embryo development. Initially, ubiquitin proteins accumulate at GV stage (Huo et al., 2004b). While whether and how ubiquitination regulates GVBD stage is unclear by now. In MI stage, SCF^*Fboxo30*^-mediated SLBP degradation regulates the chromosome condensation and segregation through ubiquitination. After MI stage, ubiquitin-conjugating enzymes UBE2C and UBE2S play roles in the escape from MII arrest (Fujioka et al., 2018). They regulate first polar body extrusion and spindle arrangement *via* APC/C^*CDC20*^ and APC/C^*CDH1*^ complex. While substrates that are ubiquitinated by UBE2C and UBE2S remain elusive. Zygote stage is the beginning of embryo development, while how ubiquitination plays roles in this stage is lack of studies in mammals. In 2-cell stage, APC/C^*CDC20*^ ubiquitinates securin and regulates sister chromatid separation. During the 4-cell to marula stage, Rnf20 controls the expression of H2B through ubiquitination and is involved in regulating preimplantation embryo development by controlling gene expression. From 8-cell to morula, ubiquitination of Suv39H1 *via* CRL4^*DCAF13*^ is important to the genome reprogramming. In morula stage, as a member of the ubiquitin C-terminal hydrolase (UCH) of deubiquitinating enzymes, UCHL1 regulates the morula compaction (Mtango et al., 2012).

Ubiquitination regulates several reproductive processes mainly by modulating protein stability. Immediately after fertilization, the degradation of maternal proteins begins and this process is mainly regulated by ubiquitination (Huo et al., 2004a). Ubiquitination is involved in reproductive processes such as oocyte maturation, pre-implanted embryo development, and sperm-oocyte binding (Figure 1). Aberrant function or regulation of ubiquitination is at the root of several developmental abnormities. Notably, the regulation of processes by ubiquitination is mainly accomplished through the binding of E3 ligases to specific targets.

### SCF Is the Largest E3 Ubiquitin Ligase Family

The S-phase kinase associated protein 1 (SKP1)-Cullin 1 (CUL1)-F-box protein (SCF) family is the largest family of E3 ubiquitin ligases (Cardozo and Pagano, 2004). The SCF complex consists of SKP1, the E3 ligase RBX1, CUL1, and an F-box protein (Zheng et al., 2002). SCF-mediated ubiquitination is known to play key roles in oocyte maturation, preimplantation embryo development, and embryonic genome activation in mammals. SCF specifically recognizes numerous substrates *via* the F-box, such as F-box and tryptophan-aspartic acid (WD) repeat domain containing 7 (FBWX7). SCF^*FBXW7*^ regulates the degradation of cyclin E, which is a key activator of the cell cycle; deletion of Fbxw7 leads to embryonic lethality (Tetzlaff et al., 2004). In addition to FBXW7, our group found FBOXO30 as a newly discovered F-box protein that was highly expressed in mouse oocytes (Jin et al., 2019). Stem-loop binding protein (SLBP) is one of the substrates of SCF^*FBOXO30*^ and regulates oocyte maturation through ubiquitination. Depletion of Fboxo30 leads to the degradation of SLBP, and the resulting excess level of histone H3 in mouse oocytes causes chromosome compaction and chromosome segregation independent of the spindle assembly checkpoint (SAC) and mouse oocyte maturation arrest at the MI stage (Jin et al., 2019). Although previous studies emphasized the crucial function of SAC on chromosome segregation (Qiao et al., 2014; Lu et al., 2017), these findings suggested a new mechanism of chromosome segregation driven by over-condensed chromosomes, rather than dependent on SAC during meiosis. Knowing the molecular mechanism gives insights into the diagnosis and therapy of infertility and early abortion in the clinic. Therefore, it will be more meaningful to examine the expression level of Fbox30 and further identify its function in female patients with infertility. Embryo genome activation is dependent on the degradation of maternal materials. As CUL1 and SKP1 are synthesized early during embryo development and are activated on day 4 and day 8 in bovine embryos (Kepkova et al., 2011), we speculate that SCF is one of the key regulators of the degradation of maternal materials during embryo genome activation.

### Other E3 Ubiquitin Ligases Involved in Embryo Development

Oocyte maturation and embryo development are very complex processes regulated by various pathways. Anaphase-promoting complex/cyclosome (APC/C) is another important E3 ubiquitin ligase that can trigger the degradation of anaphase inhibitors *via* the ubiquitin-proteasome pathway. It works with its co-activator Cdc20, which takes part in the degradation of securin (an inhibitor of separase) and finally causes sister chromatid segregation during mitosis (King et al., 1995). When Cdc20 is deleted, mouse oocyte maturation arrests at the 2-cell stage (Li et al., 2007). Moreover, CRL4 is also a common E3 enzyme. DCAF13, which is one of its substrate adaptors, is expressed from the 4-cell stage to blastocyst stage during mouse embryo development (Zhang Y. et al., 2018). CRL4 forms a complex with DCAF13 and regulates the degradation of SUV39H1 through polyubiquitination, thus playing a role in embryo development (Zhang Y. et al., 2018). Specifically, CRL4^*DCAF13*^ directs the polyubiquitination and proteasomal degradation of SUV39H1/2, and causes histone H3 demethylation and zygotic genome reprogramming. When *Dcaf13* is depleted or *Suv39h1* is overexpressed, RNA transcription in both nucleolus and nucleoplasm in the 8-cell embryo decreases (Zhang Y. et al., 2018). However, *Suv39h1* knockdown cannot rescue the embryo development arrest, which means that SUV39H1 is not the only substrate of DCAF1 through ubiquitination. Meanwhile, DCAF1 may also have CRL4-independent functions during preimplantation development. Therefore, DCAF13-dependent proteomics, which is regulated by ubiquitination, needs to be examined in future studies, so as to know more about how ubiquitination regulates preimplantation embryo development.

### Histone Proteins Regulate Chromatin Through Ubiquitination

Histone proteins can be modified by ubiquitination, and were found to play important roles in regulating chromatin architecture by as early as 1977 (Goldknopf et al., 1977). Ubiquitination of histones helps to insure the compaction and stability of chromatin. The nucleosome is the basic structural unit of eukaryotic chromosomes consisting of a 147-bp segment of DNA wrapped against a histone octamer of H2A, H2B, H3, and H4 (Fodor et al., 2010). Histones such as H2A and H2B play important roles in oocyte maturation mainly by regulating the transcription process, and the C-terminal lysine residues of H2A and H2B can be ubiquitinated. However, whether they are required for the repression of transcription is controversial (Baarends et al., 2005; Prather et al., 2009). Eskeland et al. (2010) and Endoh et al. (2012) reported that mono-ubiquitination of histone H2A (H2AK119ub1) and compaction of chromatin together regulate transcriptional repression in embryonic stem cells. Other studies suggested that loss of RING1B E3 ligase activity and H2AK119ub1 only partially disrupts polycomb recruitment (Eskeland et al., 2010; Illingworth et al., 2015). Thus, it seems that the primary role for RING1B in gene repression and early embryonic development is structural rather than enzymatic (Illingworth et al., 2015) and how H2A ubiquitination contributes to repression of its substrates is still not clear. Using intact cell MALDI-TOF mass spectrometry, researchers identified histone H2B as an oocyte meiotic maturation marker (Labas et al., 2018). Histone H2B mono-ubiquitination (H2Bub1) is mainly expressed from the late 1-cell stage to the blastocyst stage (Ooga et al., 2015). Knocking down RNF20, which is an H2B-specific ubiquitin E3 ligase, causes a decrease in the expression of H2Bub1 at the 4-cell and morula stages, and only one-third of the mouse embryos develop to the blastocyst stage (Ooga et al., 2015). Previous studies suggested that H2Bub1 regulated histone H3K4 and H3K79 methylation (Ruthenburg et al., 2007; Vermeulen et al., 2007; Lauberth et al., 2013). Interestingly, during embryo development, it seems that H2Bub1 is not the upstream regulator of H3K79me2 and H3K4me3. At present, it is not well known how H2B dynamically modulates nucleosome stability and chromatin dynamics through ubiquitination and deubiquitination during the oocyte maturation and preimplantation process (Chandrasekharan et al., 2010; Fuchs et al., 2012). These open questions have become hot topics in follow-up studies.

### Ubiquitination and Ubiquitin-Like Modifications Work Together With Other PTMs

Neddylation is a kind of ubiquitin-like modification. It is reported that inhibition of Neddyaltion led to mouse oocyte meiosis arrest at the MII stage (Yang et al., 2019). Furthermore, it is supposed to regulate oocyte maturation together with ubiquitination (Yang et al., 2019). Actually, in many cases, these processes are regulated by several modifications at the same time or sequentially rather than by only one PTM. For example, KBTBD8 is a ubiquitin ligase that is highly expressed in mouse oocytes and is essential for oocyte quality (Li et al., 2019). The mechanism by which KBTBD8 regulates oocyte maturation can be summarized as follows: KBTBD8 regulates PKM through the ubiquitination pathway and regulates Erk1/2 activity by phosphorylation (Li et al., 2019). Thus, ubiquitination and phosphorylation sequentially regulate the oocyte maturation process.

## Sumoylation During Meiosis and Embryo Development

SUMOylation is the process by which a small (∼12 kDa) SUMO protein is attached to the lysine residue of its substrates (Hendriks and Vertegaal, 2016). SUMO a ubiquitin-like modification, and most of the substrates are transcription factors (Rosonina et al., 2017). Similar to ubiquitination, SUMOylation involves a three-step cascade requiring activating enzymes (E1), conjugating enzymes (E2), and ligases (E3) (Flotho and Melchior, 2013). There are four SUMO proteins (SUMO1–4). Among them, SUMO1, SUMO2, and SUMO3 are expressed in mouse oocytes (Wang et al., 2010). During the SUMOylation process in mammals, SUMO proteins are cleaved by sentrin-specific proteases (SENPs) to expose the C-terminus (Eifler and Vertegaal, 2015). Then an E1 activating enzyme (SAE1) activates the SUMO polypeptide in an ATP hydrolysis-dependent manner. SUMO is transferred to the only E2 conjugating enzyme, Ubc9. Finally, the E3-ligating protein attaches SUMO to its specific substrates (Swatek and Komander, 2016). SUMO predominantly accumulates in the nucleus and plays a crucial role in many biological processes such as gene expression, DNA damage responses, chromosomal stability, and DNA integrity (Di Bacco et al., 2006; Schwertman et al., 2016). Furthermore, SUMOylation is essential during meiosis and embryo development.

### Ubc9 Involves in Mammalian Oocyte Maturation as E2 Conjugating Enzyme

As mentioned above, the E2 conjugating enzyme Ubc9 is indispensable for SUMOylation. It has been reported that loss of Ubc9 in chick cells causes chromosome damage and eventually results in apoptosis (Hayashi et al., 2002). The mechanism of how SUMOylation, and especially Ubc9, regulates oocyte maturation and embryo development is not fully understood. Loss of *Ubc9* causes embryonic lethality due to defects in chromosome segregation and lack of integrity of the nucleus in mouse embryos (Nacerddine et al., 2005). Inhibition of Ubc9 at the GV stage causes meiotic maturation arrest and spindle disorganization (Nacerddine et al., 2005). Another study recently demonstrates that when Ubc9 is added to the oocyte maturation medium, the level of SUMO-1 decreases, thereby inhibiting the first polar body exclusion and reducing the oocyte maturation rate (Xu et al., 2020). It is suggested that CaMKII is the substrate of SUMO-1 during embryo development but has not been surely identified yet in this study (Xu et al., 2020). In conclusion, Ubc9-mediated SUMOylation affects meiosis and preimplantation development mainly by regulating spindle organization and chromosome segregation.

### E3 SUMO Ligases and the Substrates

In addition to Ubc9, E3 ligases and their specific substrates are also crucial for oocyte maturation and embryo development. Polo-like kinase 1 (PLK1) is one of the main substrates of E3 ligase and undergoes SUMOylation during oocyte maturation. It has a non-catalytic region at the C-terminus containing the Polo-box domain, which has been implicated in phosphorylation of serine/threonine residues (Lee et al., 1998) and functions during chromosome segregation, spindle formation, and cytokinesis (Petronczki et al., 2007; Hu et al., 2012; Ruan et al., 2012; Zhu et al., 2013). Many studies have identified the importance of PLK1 in centrosome maturation, checkpoint recovery, and spindle assembly in mitosis and meiosis (Pahlavan et al., 2000; Tong et al., 2002; Haren et al., 2009). However, few studies have investigated whether it takes part in SUMOylation. It has been reported that SUMO1-mediated spindle assembly and cytokinesis are regulated by SUMOylation of PLK1 after the GVBD stage (Feitosa et al., 2018). During the MI and MII stages, SUMO2 and SUMO3 are co-localized to the centromeric region in mouse oocytes (Feitosa et al., 2018). This suggests that PLK1 modification by SUMO2/3 regulates the attachment of chromosomes to the spindle during mouse meiosis.

Maternal protein PIASy (inhibitor of activated STATy) is another important E3 SUMO ligase. During embryo development, overexpression of PIASy causes embryo arrest at the 2-cell stage because of abnormal chromosome segregation and impaired zygotic transcription (Higuchi et al., 2019). SENP7, a SUMO poly-chain editing enzyme, plays a key role in heterochromatin integrity and binds to chromatin by interacting with HP1a (Hickey et al., 2012; Garvin et al., 2013; Romeo et al., 2015). During mitosis, loss of SENP7 increases the SUMOylation of HP1a and inhibits the expression of genes such as Rad51 and BRCA1, which are related to DNA damage repair (Maison et al., 2011). During meiosis, depletion of SENP7 inhibits polar body extrusion (Huang et al., 2017). It is speculated that SENP7 can cleave SUMO2/3 chains, thereby preventing the ubiquitination-mediated degradation of γ-tubulin, and finally causing meiosis arrest (Huang et al., 2017). These findings provide novel targets for developing the therapies and diagnosis of infertility and improving human fertility in clinic.

## Parylation Is a Novel Marker of Oocyte and Embryo Quality

PARylation involves the transfer of ADP-ribose moieties from co-enzyme NAD + to aspartate, glutamate, serine, and lysine (Kraus and Lis, 2003; Martello et al., 2016), and is a pivotal PTM playing important roles in many biological processes including DNA repair, cell apoptosis, and transcriptional regulation (D’Amours et al., 1999). PARylation occurs rapidly after DNA damage and is catalyzed by PARPs (Gibson and Kraus, 2012), which are a 17-member family and covalently attach ADP-ribose to their substrates (Hottiger, 2015; Wei and Yu, 2016). Most PARPs only transfer a single mono-ADP-ribose onto their substrates, while PARP1, PARP2, PARP5A (also known as tankyrase 1), and PARP5B (also known as tankyrase 2) are able to add additional ADP-ribose molecules and form linear or branched chains of PAR (Bai, 2015). Though it is accepted that PARylation is crucial for oocyte and embryo development, little is known about its underlying mechanisms.

PARylation is essential for oocyte asymmetric division and preimplantation embryo development. It is reported that PAR is enriched at the cortex of the mouse oocyte (Xie et al., 2018). When the cortical PAR is removed by spindle exchanging, the asymmetric division is disrupted (Xie et al., 2018). During embryo development, the level of PARylation dynamically changes, especially in the first hour after fertilization. PARPs accumulate to high levels on the condensed oocyte chromosomes; the level of PARPs decreases 2 h later, and only a diffuse signal is detected in the cytoplasm of telophase- and pronuclear-stage embryos (Imamura et al., 2004). Consistent with these observations, another study reports that the transcript level of PARP1 transiently increases when fertilization occurs, decreases at the late one-cell stage, and is maintained until the blastocyst stage (Lee et al., 2016a). This indicates that PARylation occurs transiently from metaphase to interphase, and that spindle-associated PARP may be activated during this time. As there is no evidence that DNA repair occurs in activated oocytes, PARylation may play roles independent of its DNA damage repair capacity.

It is also suggested that PARylation may regulate autophagy during early embryo development (Lee et al., 2016a). It is speculated that autophagy is a positive regulator and can improve the quality of oocytes and embryos (Song et al., 2012, 2014). When PARylation is inhibited, the expression of autophagy-related genes is significantly decreased and the developmental competence and quality of porcine embryos are diminished (Lee et al., 2016a). During blastocyst formation, inhibition of PARylation selectively suppresses autophagic degradation of ubiquitinated proteins (Lee et al., 2016a). The effect of PARylation on autophagic degradation was subsequently found to be mediated by the mTOR pathway (Lee et al., 2016b). Specifically, PARylation, which activates AMPK and negatively regulates the mTOR pathway (Zhou et al., 2013; Chen et al., 2015), causes autophagy during the blastocyst formation process in porcine embryos by decreasing p70S6K accumulation and thus inhibiting the activity of mTORC1 (Lee et al., 2016b). In this way, PARylation accurately regulates the embryo development through MAPK and mTOR pathways. Moreover, treatment of mice with a PARylation inhibitor causes subfertility, with embryos arresting at the pronuclear envelope breakdown stage (Osada et al., 2010). PARP1-deficient mice are fertile (Masutani et al., 1999), while PARP1 and PARP2 double mutant embryos die early in development at the onset of gastrulation (Ménissier de Murcia et al., 2003). It is clear that PARylation does play a role in reproductive processes, but the concrete mechanism is not clear by now.

## Conclusion

As discussed above, we concluded main functions of phosphorylation, ubiquitination, SUMOylation and PARylation during oocyte maturation and embryo development (Table 1). Besides the PTMs we reviewed above, a small number of studies found that other PTMs, such as glycosylation and acetylation, also involved in oocyte maturation and embryo development. Pannexin1 is a kind of glycoproteins encoded by *PANX1*. With a diagnosis of primary infertility of unknown causes, a four-generation family was included in a recent study (Sang et al., 2019). Researchers identified four kinds of mutations of *PANX1* by whole-exome sequencing and observed that mutant GLY1 (one glycosylation form of PANX1) caused oocyte death because of aberrant channel activation (Sang et al., 2019). Other studies showed that acetylation was involved in regulating meiotic progression and maternal-to-zygotic transition (Wang H. et al., 2019; Wang H.H. et al., 2019; Zhang et al., 2019). Moreover, it was indicated that lysine acetylation in polycystic ovary syndrome (PCOS) granulosa cells affected the ovarian metabolic microenvironment and damaged the oocyte quality and embryo development (Min et al., 2020). The acetylation level of acetyl-CoA acetyltransferase 1 was negatively correlated with the fertilization rate and the ratio of transferable embryos (Min et al., 2020). This study revealed ACAT1 as a metabolic regulator functioning through acetylation, and provided a novel target for future investigations and clinical therapy of PCOS.

**TABLE 1:** Main PTMs of oocyte maturation and early embryo development.

	**Protein name**	**Main function**	**Related factors**	**References**
phosphorylation	Histone H3	Chromosome condensation, homologous chromosome segregation	MEK1/2, ERK1/2	Huang et al., 2018; Wang et al., 2006; Besterman and Schultz, 1990
	MAPK	Spindle formation, kinetochore and microtubules	Toxines(bisphenol AF, HT-2, zearalenone)	Villeneuve et al., 2012; Ding et al., 2017; Zhu et al., 2014
	cofillin	Spindle orgznization, actin assembly	Protein kinase D subfamily	Zhang Y. et al., 2018
ubiquitination	cyclinE	embryo development	SCF^*FBW7*^ comples	Tetzlaff et al., 2004
	SLBP	Chromosome condensation, chromosome segregation	SCF^*FBOXO30*^ complex, histone H3	Jin et al., 2019
	Histone H2A	Transcriptional repression regulation in early embryonic development	RING1B E3 ligse	Eskeland et al., 2010; Illingworth et al., 2015
	histone H2B	regulation of meiosis and preimplantation development	RNF20, Bre1p	Ooga et al., 2015; Labas et al., 2018
	securin	Sister chromatid segregation	APC/C complex, CDC20	King et al., 1995; Li et al., 2007
	SUV39H1	Zygotic genome reprogramming	CDAF13, CRL4	Zhang Y.-L. et al., 2018
NEDDylation (ubiquitin-like PTM)	Emi1	spindle assemby checkpoint	CUL1, APC/C complex	Yang et al., 2019
SUMOylation	Ubc9	seindle organization, extrusion of first polar body	SUMO1	Xu et al., 2020
	PLK1	Centrosome matuation, spindle assemby	SUMO1/2/3	Feitosa et al., 2018; Tong et al., 2002
	PIASy	Chromosome segregation; zygotic transcription	SUV39H1	Higuchi et al., 2019
	Hp1a	Polar body exclusion	γ-tubulin, SUMO2/3	Huang et al., 2017; Romeo et al., 2015
PAPylation	PARPs	Oocyte chromosome condensation, asymmetric division, embryo development, autophagy	MAPK pathway, mTOR pathway	Xie et al., 2018; Imamura et al., 2004; Lee et al., 2016a; Zhou et al., 2013; Chen et al., 2015

High-quality oocytes are the foundation for fertilization and embryo development. Any error in oocyte maturation may cause deficiencies including infertility, abortion, and birth defects. The regulation of oocyte maturation and embryo development is incredibly complex. Because of the lack of transcription, PTM appears to be indispensable for oocyte maturation and early embryo development (Chen et al., 2011). Though PTMs have been investigated for decades, most studies have been done in non-mammalian systems such as Xenopus oocytes, because of the large number of maternal proteins and limited techniques for researching. As we described in this review, PTMs play particularly important roles in the regulation of chromosome condensation, spindle morphology, and spindle segregation through different modifications. It is worth noting that the dynamic changes of each PTM during different stages seem to be crucial and not fully explored yet. Furthermore, it seems that the oocyte maturation and preimplantation development are regulated by more than one kind of PTM in many cases. Different modifications may work together at the same time or sequentially. So, with the improvement of biological research methods and techniques, future research will give new insights into the complex mechanism of oocyte maturation and embryo development. Knowing the mechanisms of how PTMs regulate reproductive processes will provide new targets for the clinical diagnosis and treatment of infertility. Moreover, new contraceptive drugs are expected to be investigated in the future.

## Author Contributions

All authors listed have made a substantial contribution to the work and wrote the manuscript.

## Conflict of Interest

The authors declare that the research was conducted in the absence of any commercial or financial relationships that could be construed as a potential conflict of interest.
